# Multiple and Multidirectional Fissure Bleedings in a Patient With a Spontaneous Isolated Dissection of the Iliac Artery

**DOI:** 10.7759/cureus.38374

**Published:** 2023-05-01

**Authors:** Sei Komatsu, Satoru Takahashi, Chikao Yutani, Mitsuhiko Takewa, Tomoki Ohara, Kazuhisa Kodama

**Affiliations:** 1 Cardiovascular Center, Osaka Gyoumeikan Hospital, Osaka, JPN

**Keywords:** aortic injury, non-obstructive general angioscopy, aortic dissection, angioscopy, aorta, fissure bleeding, iliac artery, isolated aortic dissection of the iliac artery

## Abstract

A 63-year-old man with a history of hypertension and dyslipidemia on medication was found to have an enlargement of an asymptomatic iliac artery aneurysm with an ulcer-like projection on computed tomography angiography. The longer and shorter diameter of the right iliac was increased from 24.0 × 18.1 mm to 38.9 × 32.1 mm over four years. Preoperative non-obstructive general angiography revealed multiple, multidirectional fissure bleedings. Fissure bleedings were found where computed tomography angiography appeared normal at the aortic arch. He was diagnosed with spontaneous isolated dissection of the iliac artery and was treated successfully with endovascular treatment.

## Introduction

Spontaneous isolated dissection of the iliac artery is rare [1]. Isolated aneurysms of the iliac arteries account for less than 2% of aortic aneurysms [2]. Isolated iliac artery aneurysms are related to a significant risk of rupture and death [3]. Clinical data suggest that endovascular aortic repair (EVAR) improves perioperative outcomes and is associated with comparable long-term survival rates to open surgery [4]. Only a few invasive imaging methods are available to diagnose or guide the treatment of aortic aneurysm or dissection. Intravascular ultrasound (IVUS) has been attempted as a less invasive and potentially more effective option than aortography [5]. Non-obstructive general angioscopy (NOGA) is used for internal exploration of the aorta [6]. NOGA can safely detect spontaneously ruptured aortic plaques and injuries of aortic dissection [7]. Recently, determining the position of a stent graft by the aortic injury seen by NOGA has been proposed [8]. Here, we report the case of a patient with an iliac dissecting aneurysm shown as isolated, multiple, and multidirectional injuries by NOGA.

## Case presentation

**Figure 1:**
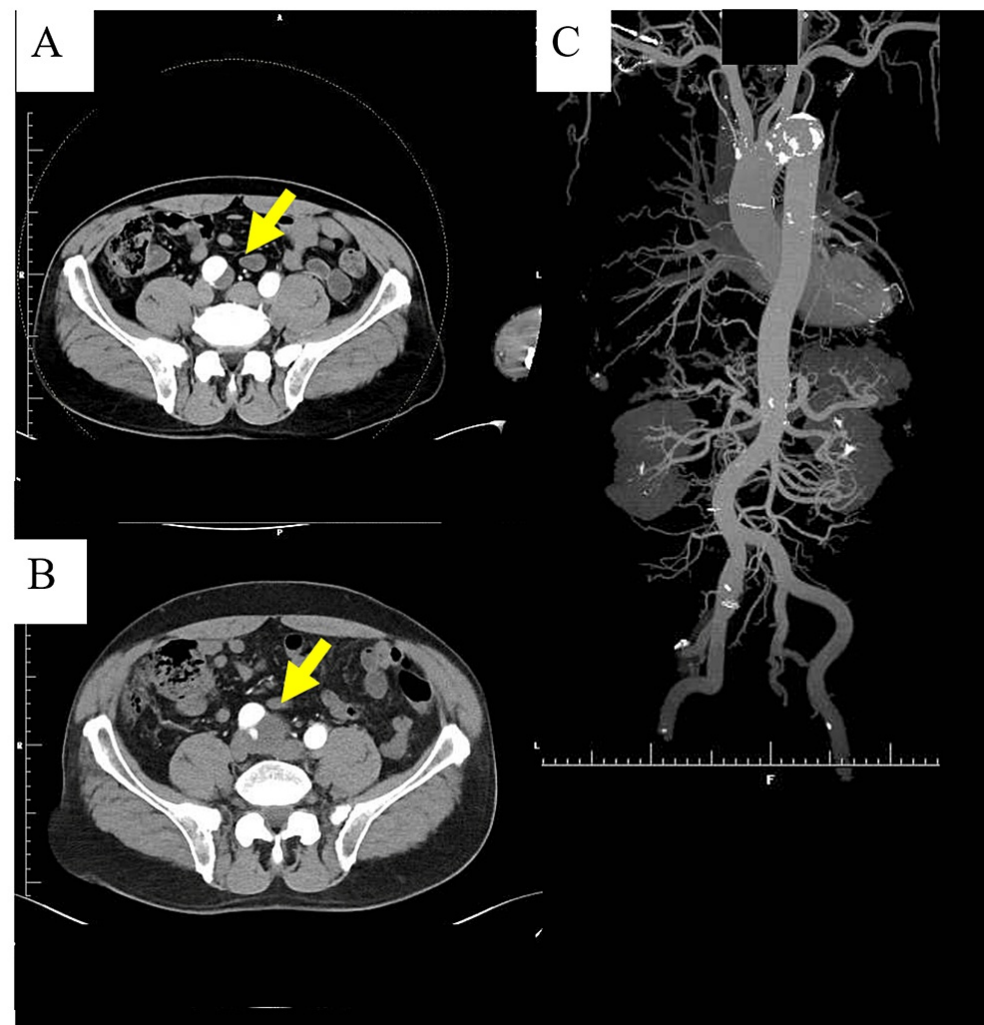
A series of computed tomography angiography images. A. The iliac artery aneurysm four years ago (an arrow). B. The iliac artery aneurysm on admission (an arrow). C. Three-dimensional reconstruction of the aorta. Calcification was found predominantly in the aortic arch.

A 63-year-old man, taking medication for hypertension and dyslipidemia, was found to have an asymptomatic enlargement of the right iliac aneurysm by computed tomography during a routine investigation. He had been prescribed 5 mg of amlodipine, 5 mg of rosuvastatin, and 10 mg of ezetimibe for seven years. He had no history of smoking, trauma, or heavy exercise. The longer and shorter diameter of the right iliac aneurysm was 24.0 × 18.1 mm four years ago (Figure 1A and Video 1). The size of the aneurysm increased to 38.9 × 32.1 mm with an ulcer-like projection (Figure 1B and Video 1). A low-density area at the aneurysm was also enlarged. Computed tomography angiography revealed no stenosis or occlusion in the coronary arteries. Calcification was found in the aortic arch; however, few were found in the abdominal and iliac arteries (Figure 1C).

**Video 1:** A series of axial images of computed tomography aortography four years ago, non-enhanced computed tomography on admission, and computed tomography aortography on admission.

Digital subtraction angiography demonstrated abnormal blood flow from the right common iliac artery into the aneurysm (Video 2).

**Video 2:** Digital subtraction angiography for the dissecting aneurysm of the right iliac artery. Abnormal blood flow from the right common iliac artery into the aneurysm was found.

**Figure 2:**
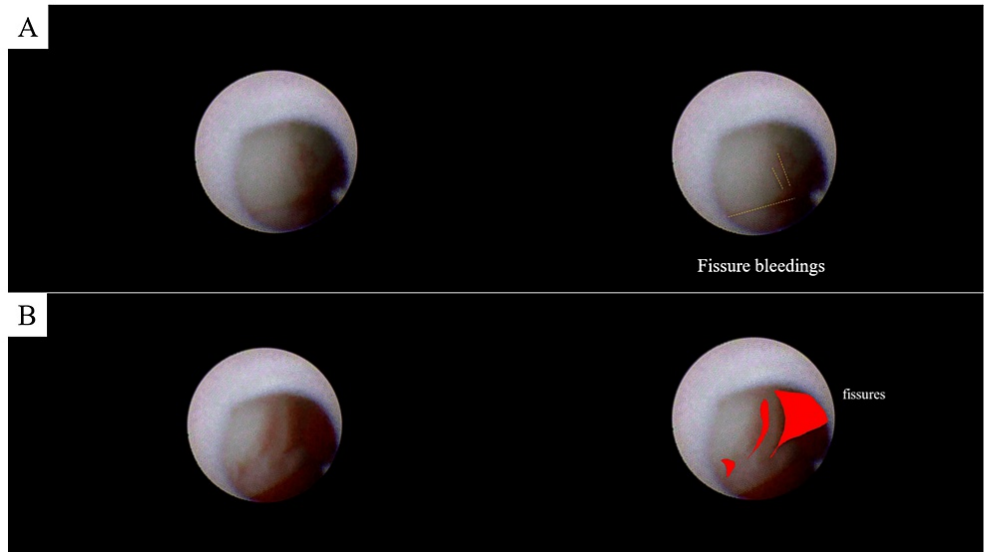
Angioscopic images of the iliac artery aneurysm without (left) and with captions (right). A. Multiple and multidirectional fissure bleedings. B. Bleedings from large fissures.

The aneurysm at the right common iliac artery was diagnosed with the dissecting aneurysm. NOGA was performed for the whole aorta [6]. The NOGA system consisted of a VISIBLE Fiber (FT-203F, Fiber Tech Co. Ltd., Tokyo, Japan), a ﬁber imaging system, and a console (Intertec Medicals Co. Ltd., Osaka, Japan). Multiple and multidirectional fissure bleedings were found on a white aortic wall in the dissecting aneurysm of the right common iliac artery (Figure 2).

**Figure 3:**
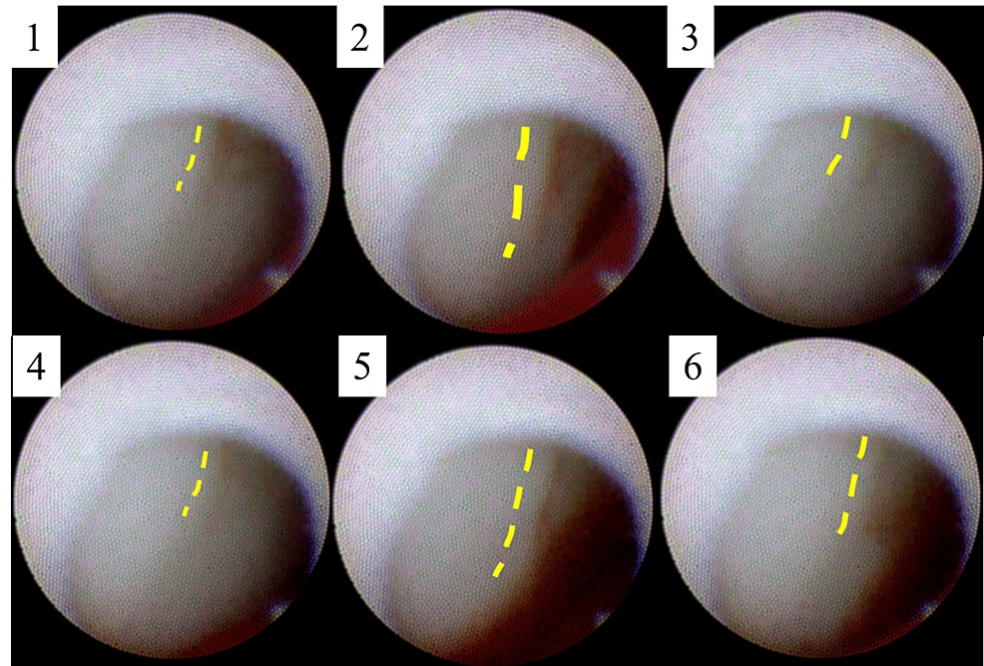
A series of angioscopic images at an interval of 10 frames for a fissure. A video was recorded at 30 frames per second. A red line at the fissure becomes thicker and thinner, suggesting that blood flows to and fro between the aortic wall and subintima (yellow dotted lines).

These fissures might be entry tears. One representative fissure showed a red line at the fissure becoming thicker and thinner (Figure 3).

**Figure 4:**
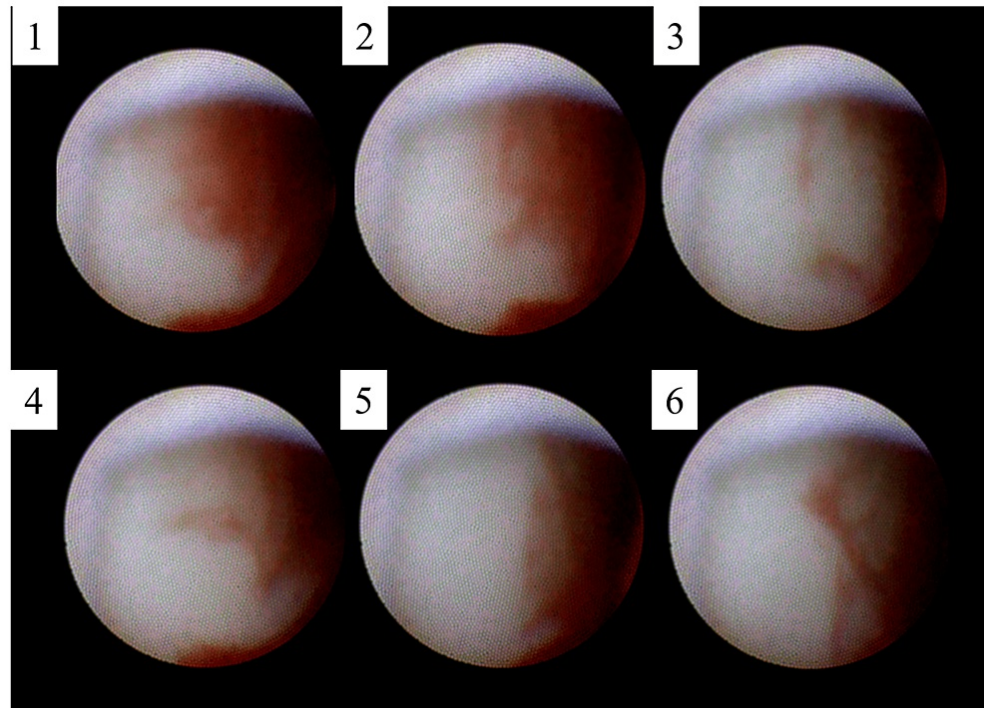
A series of images for an intramural hematoma similar to the wall painting near the fissure bleedings shown by non-obstructive general angioscopy. The intramural hematoma changed its shape.

This may mean that positive and negative pressures were alternately applied between the aortic wall and subintima. In addition, a tiny fluttering flap and an intramural hematoma that changed its shape near the fissure bleeding similar to previous findings were detected (Figure 4 and Video 3) [7].

**Video 3:** Angioscopic video of the dissecting aneurysm of the right iliac artery. Multiple and multidirectional fissure bleedings, a tiny fluttering flap, and an intramural hematoma were detected.

**Figure 5:**
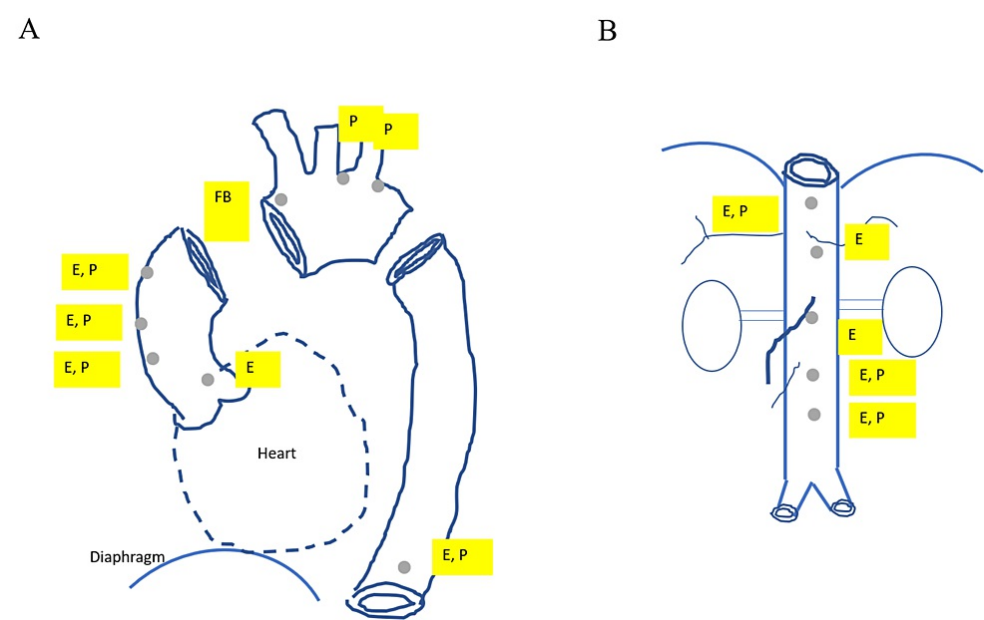
A schematic image of spontaneous ruptured aortic plaques detected by non-obstructive general angioscopy. A. The thoracic aorta. B. The abdominal aorta P: puff rupture; E: erosion; FB: fissure bleeding

The change in the intramural hematoma did not seem to be associated with the aortic blood flow. Aortic atherosclerosis did not seem severe except for the right common iliac artery. Spontaneously ruptured aortic plaques such as puff rupture that scattered like puff and erosion were detected in the descending aorta, the aortic arch, and the abdominal artery. At the aortic arch, there was no sign of aortic dissection by computed tomography (Video 1); however, two fissure bleedings were detected by NOGA (Figure 5 and Video 4).

**Video 4:** Angioscopic video of the dissecting aneurysm of the aortic arch. Two fissure bleedings were detected.

He underwent EVAR for the isolated dissecting aneurysm of the right common iliac artery. The injuries at the aortic arch were medically followed as they were unsatisfied with the operation indication. He remained uneventful for one year.

## Discussion

Iliac artery aneurysm is associated with a significant risk of rupture when it reaches a considerable size [3]. Aortic aneurysm management, a life-threatening condition, has undergone significant changes in the last two decades. There is a greater inclination toward EVAR than open surgical repair [3,9]. However, with EVAR, the information regarding the underlying cause of the disease becomes even more limited compared to surgery. IVUS can be reliably used in the sizing and planning of the EVAR stent graft, along with complementary non-contrast imaging [9]. However, it is challenging to identify the damage to the blood vessels that may cause the disease using IVUS because it cannot demonstrate aortic plaques more precisely than NOGA [6]. The predisposing risk factors of spontaneous isolated iliac artery dissection include traumatic and non-traumatic, such as cystic medial degeneration and fibromuscular dysplasia [10,11]. Our patient did not have a history of trauma and no findings suggestive of diseases such as cystic medial degeneration. While penetrating atherosclerotic ulcer permits blood to penetrate the aortic media, the presence of atherosclerotic scarring in the aorta usually restricts blood accumulation, frequently leading to a confined dissection or pseudoaneurysm [12]. The aortic aneurysm was thought to be the cause of the iliac artery aneurysm using digital subtraction angiography and NOGA. NOGA demonstrated multiple fissure bleedings. Previously, we reported a patient with chronic communicating aortic dissection with multiple fissures [7]. Multidirectional fissures in aortic dissection are rarely reported. Fissure bleedings have been found in non-communicating-type aortic dissection [13]. A low-density area in the aorta on computed tomography aortography images does not mean a thrombus [14]. In NOGA, the primary technique involves injecting low-molecular-weight dextran into the area between the catheters and the fiber to achieve a visual field expansion; the diluted blood results in a broader field of view [15]. When observing the aortic wall with NOGA, the field of view appears transparent. The blood flowing into the aortic lumen from the fissure of the aortic wall remains visible. In such cases, the appeared blood mixes with the transparent liquid and gradually disappears (Video 3). However, as shown in Figure 3, the blood remaining in the fissure did not disappear entirely but became thinner when observing the fissure continuously. This phenomenon is because of the transparent liquid entering the subintimal layer through the fissure, causing the blood attached to the fissure to disappear without being swept away. Angioscopically, bidirectional blood flow was seen between the aortic lumen and the subintimal layer through the fissure. Fissure bleeding was not detected in other parts of the aorta, except for the aortic arch. Mild spontaneously ruptured aortic plaques were identified throughout the aorta. Thus, the patient’s iliac artery aortic dissection was thought to be isolated. Here, because the injury was limited to an aneurysm, the patient had a favorable outcome by occluding it with stent graft surgery. It has been reported that NOGA can confirm the injuries during the intervention of aortic dissection and achieve good results by covering them with EVAR [8]. Although NOGA is an invasive method, it is a safe procedure [16-18].

## Conclusions

Although spontaneous isolated dissection of the iliac artery is rare, it has a potential risk of aneurysmal development, rupture, and death. The patient’s aorta did not cause remarkable injuries except for the multiple and multidirectional fissure bleedings in the iliac artery. While accumulating cases is necessary, NOGA provides information about the underlying cause of aortic dissection before EVAR.
